# Synergistic Mechanistic Insights into Anti-T2DM Benefits of *Lentinula edodes*: A Peptide- and Polysaccharide-Based Network Pharmacology and Molecular Docking Study

**DOI:** 10.3390/foods15030453

**Published:** 2026-01-27

**Authors:** Hui-Ke Ma, Lei Meng, Liang Shen, Hong-Fang Ji

**Affiliations:** 1 School of Life Sciences, Ludong University, Yantai 264025, China; 13173438702@163.com; 2 Institute of Food and Drug Research for One Health, Ludong University, Yantai 264025, China; 6315@ldu.edu.cn; 3 School of Food Engineering, Ludong University, Yantai 264025, China

**Keywords:** *Lentinula edodes*, peptides, polysaccharides, T2DM, network pharmacology, molecular docking

## Abstract

In recent years, dietary intervention has garnered significant attention for T2DM prevention and adjunctive treatment. *Lentinula edodes* (commonly known as shiitake mushroom), a common edible fungus, has been demonstrated to improve T2DM, primarily attributed to its main bioactive components like peptides and polysaccharides, while their synergistic characteristics are still not fully explained. Therefore, this study investigated the anti-T2DM molecular mechanisms of *L. edodes* peptides and polysaccharides by integrating network pharmacology and molecular docking. First, systematic searches of the PubMed and HERB databases using keywords such as “*Lentinula edodes* peptides”, “*Lentinula edodes* polysaccharides” and “T2DM” and “*Lentinula edodes*/shiitake mushroom” yielded 25 peptides and 14 polysaccharides. Second, network pharmacology analysis revealed 541 common interaction targets between these peptides/polysaccharides and T2DM. Topological analysis further identified nine core targets: ESR1, MAPK1, AKT1, SRC, EGFR, STAT3, JUN, PIK3CA, and PIK3R1. Third, pathway enrichment analysis showed that these core targets were significantly enriched within the PI3K-Akt signaling pathway and the AGE-RAGE signaling pathway in diabetic complications, suggesting potential anti-T2DM effects through regulation of these key pathways. Finally, molecular docking validation ensured strong binding affinities between peptides/polysaccharides and some core targets, with particularly prominent binding capacities observed for peptides VF and LDELEK with EGFR; peptides KIGSRSRFDVT, LDYGKL, and EDLRLP along with polysaccharides D-glucan and β-glucan with PIK3CA; and peptide DVFAHF with PIK3R1. In summary, this study revealed that *L. edodes* peptides and polysaccharides may exert synergistic anti-T2DM effects via the regulation of key signaling pathways, including the PI3K-Akt signaling pathway, EGFR tyrosine kinase inhibitor resistance, and the AGE-RAGE signaling pathway in diabetic complications, through their actions on critical targets such as ESR1, PIK3CA, and PIK3R1. These results offer a synergistic mechanism for the anti-T2DM effect of *L. edodes*, which could be helpful for the development of functional foods and drugs derived from *L. edodes*.

## 1. Introduction

Type 2 diabetes mellitus (T2DM) is a chronic metabolic disorder, contributing to about 90% of the diabetic cases globally, and their prevalence is steadily increasing annually [1,2]. Its chief pathologic characteristic is defined by absolute or relative deficiency of endogenous insulin and disturbed utilization of insulin by the body [3,4]. Chronic hyperglycemia may trigger a wide array of severe and life-threatening complications, including non-alcoholic fatty liver disease (NAFLD) [1], cardiovascular disorders, and dyslipidemia [5]. The first-line clinical treatments for T2DM at present primarily include metformin [6], sulfonylureas, and glucagon-like peptide-1 receptor agonists [7]. However, the abovementioned drugs often possess a significant assemblage of side effects, like gastrointestinal discomfort, weight gain, and hypoglycemia [8,9,10,11], which seriously reduce patients’ treatment compliance and limit long-term efficacy in glycemic control. Thus, an effective and safe intervention for T2DM has become an urgent need. In recent years, dietary interventions have attracted researchers’ attention for T2DM prevention and/or treatment due to their extensive availability and fewer side effects [12]. It is well known that the adoption of healthy dietary habits could significantly decrease the risk of developing T2DM and promote glycemic control and metabolic health among diabetic patients [2,13,14]. Among the various functional foods or natural products, different bioactive components, including peptides, polysaccharides, and polyphenols, have shown the ability to improve glucose homeostasis. Their functions are chiefly the enhancement of insulin sensitivity, the modulation of inflammation, and the balance of the gut microbiota [15,16,17,18,19]. Hence, it is stated that an in-depth understanding of natural food ingredients with hypoglycemic potential and their mechanisms of action may not only illuminate the scientific explanation for dietary intervention but also provide new insights into developing safe and effective nutritional intervention strategies for T2DM.

As one of the most popular edible fungi with rich nutritional value, *Lentinula edodes* has been reported to possess significant natural medicinal potential [20]. It serves as a rich source of various bioactive substances and nutritional factors [21]. Many studies have discovered that the main constituents of *L. edodes*, such as peptides and polysaccharides, possess various physiological functions like immunomodulation, anti-inflammatory [22] and anticancer activities [23,24]. Moreover, both *L. edodes* peptides and polysaccharides have been found to possess blood glucose-lowering potential according to some studies [25,26,27]. However, there have been limited studies conducted on the synergistic effect between these two bioactive components against T2DM and their complex molecular mechanisms. Therefore, more research is required on this topic.

Traditional experimental approaches such as immunohistochemistry and Western blots are often time-consuming and labor-intensive, with many technical difficulties in the study of complex biological mechanisms. In this respect, network pharmacology, as an emerging systems biology approach, has become an essential tool in drug target identification and in determining the interaction between drugs and proteins [28]. It integrates systems biology, genomics, proteomics, and big data analysis to construct multidimensional “drug-component-target-disease” networks through computational modeling. This enables the prediction of active compound components and their potential disease targets at the systems level [29,30]. The network pharmacology methodology not only helps further understand the synergy mechanism of *L. edodes* peptides and polysaccharides in combating T2DM but also offers a promising perspective for further development of new hypoglycemic drugs based on these compounds. Such an approach provides new strategies for the long-term management of T2DM.

**Figure 1:**
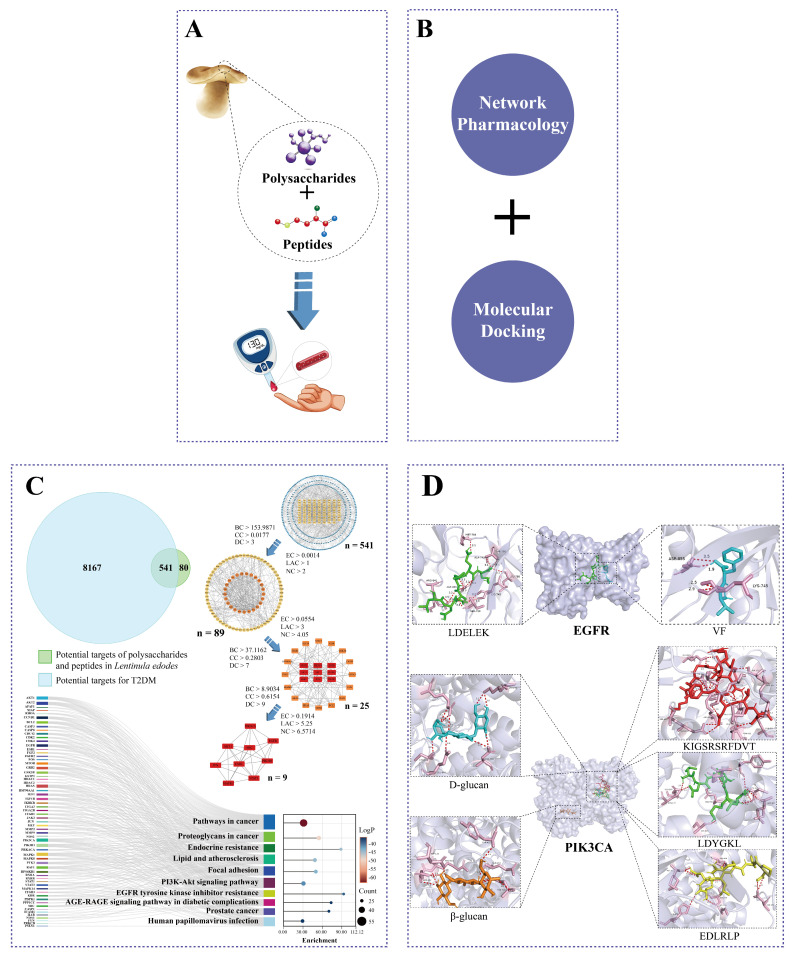
Framework of the synergistic mechanistic study on anti-T2DM benefits of *L. edodes*. (**A**) *L. edodes* peptides and polysaccharides improve T2DM. (**B**) Network pharmacology and molecular docking were used in this study. (**C**) Target screening and KEGG analysis. (**D**) Visual analysis of docking results between both *L. edodes* peptides and polysaccharides and core target molecules.

Therefore, the current study incorporated network pharmacology and molecular docking techniques to elucidate the mechanisms of *L. edodes* peptides and polysaccharides against T2DM (Figure 1). The possible action targets of *L. edodes* peptides and polysaccharides against the T2DM-related targets were firstly identified based on searches from multiple databases. Subsequently, network pharmacology analysis identified 541 common interaction targets, which were further refined through topological analysis to select nine core targets: ESR1, MAPK1, AKT1, SRC, EGFR, STAT3, JUN, PIK3CA, and PIK3R1. Enrichment analysis indicated that these targets were significantly enriched with pathways that were pivotal to insulin resistance and glucose metabolism, notably the PI3K-Akt signaling pathway, the AGE-RAGE signaling pathway in diabetic complications, and EGFR tyrosine kinase inhibitor resistance. Finally, molecular docking was employed to confirm the binding ability or modes of interaction between these bioactive components (peptides and polysaccharides) and these nine core targets, thereby elucidating their possible mechanisms for improving T2DM.

## 2. Materials and Methods

### 2.1. Screening of L. edodes Peptides and Polysaccharides

To obtain information related to *L. edodes* peptides and polysaccharides, this study systematically searched multiple databases. In PubMed, comprehensive searches were conducted using the keywords “*Lentinula edodes*/shiitake mushroom + diabetes”, “*Lentinula edodes*/shiitake mushroom + peptides”, and “*Lentinula edodes*/shiitake mushroom + polysaccharides”. Additionally, a supplementary search was performed in the HERB database using the keyword “*Lentinula edodes*/shiitake mushroom”.

### 2.2. Predicting Targets of L. edodes Peptides and Polysaccharides

Firstly, the 2D structure of an *L. edodes* peptide was drawn using the ChemDraw software, while the 2D structure of an *L. edodes* polysaccharide was retrieved from the PubChem database. Next, the *L. edodes* peptide structure was submitted to both the PharmMapper database (Z′-score ≥ 0.5) and the SwissTargetPrediction database (probability > 0) for target prediction. At the same time, the *L. edodes* polysaccharide structures were submitted to the SwissTargetPrediction database (Probability > 0) for analysis. Finally, the predicted targets were assessed against the UniProt ID database, and redundant targets were removed to ensure target specificity.

### 2.3. Identification of Therapeutic Targets for T2DM

To extensively identify the disease targets associated with T2DM, this study searched a number of disease databases. Keyword searches were conducted using “T2DM”, “type 2 diabetes mellitus”, and “Diabetes Mellitus, Type 2” in the Online Mendelian Inheritance in Man (OMIM), Therapeutic Target Database (TTD), DrugBank, and GeneCards (relevance score ≥10) databases. All the target data collected was consolidated, and duplicates were removed, yielding a non-redundant set of potential therapeutic targets for subsequent analysis.

### 2.4. Protein–Protein Interaction Network Construction and Analysis

A cross-analysis was conducted by comparing the predicted targets of *L. edodes* peptides and polysaccharides with the disease targets of T2DM. Their common targets were visualized using a Venn diagram to find the possible targets for the anti-T2DM effects of *L. edodes* peptides and polysaccharides. The common targets were imported into the STRING database for the construction of a protein–protein interaction (PPI) network. The minimum required interaction score was assigned as the highest confidence level of 0.900, and isolated nodes were removed. Then, the obtained network was exported as a TSV file and was further loaded into the Cytoscape 3.10.3 software using the CytoNCA 2.1.6 plugin for the purpose of topological analysis. By iterative screening, only nodes surpassing the median value in all six topological metrics, including Betweenness Centrality (BC), Closeness Centrality (CC), Degree Centrality (DC), Eigenvector Centrality (EC), the Local Average Connectivity-based method (LAC), and Network Centrality (NC), were retained to identify the core targets.

### 2.5. Functional Enrichment and Network Analysis

In order to elucidate the underlying mechanisms of synergy between the *L. edodes* peptides and polysaccharides in improving T2DM, we carried out a systematic enrichment analysis with the Metascape platform based on 89 cross-targets identified from the initial screening process. The analysis encompassed Gene Ontology (GO) enrichment involving the biological process (BP), molecular function (MF), and cellular component (CC) categories, as well as a Kyoto Encyclopedia of Genes and Genomes (KEGG) analysis. The species was generalized as “Homo sapiens” for all analyses. To comprehensively elucidate the mechanism of action, we further constructed an integrated “peptide/polysaccharide-pathway-disease” network so as to systematically reveal the multi-target, multi-pathway synergistic mechanism by which *L. edodes* peptides and polysaccharides combat T2DM.

### 2.6. Molecular Docking Analysis

For analyzing the interactions between both *L. edodes* peptides and polysaccharides and the core targets identified through screening, we conducted molecular docking analysis between the nine core targets previously identified and 25 *L. edodes* peptides, as well as 14 *L. edodes* polysaccharides. The 2D structures of *L. edodes* peptides were constructed using ChemDraw, while the 2D structures of *L. edodes* polysaccharides were retrieved from the PubChem database. All molecular structures were converted to 3D structures via Chem3D and optimized through energy minimization. The structures of the core targets’ proteins were obtained from the PDB database. After removing water molecules and native organic molecules using the PyMOL 2.4.0a0 software, their structures were hydrogenated and optimized using the AutoDock 1.5.7 software. The processed peptides and polysaccharides served as ligands, while the target proteins acted as receptors when carrying out molecular docking by the AutoDock Vina 1.1.2. And the specific parameters for molecular docking are briefly outlined in Supplementary Table S1. Finally, docking results were visualized with PyMOL 2.4.0a0, and a binding affinity heatmap was prepared using the GraphPad Prism software.

## 3. Results

### 3.1. Retrieval and Acquisition of L. edodes Peptides and Polysaccharides

Through systematic searches of the PubMed database, we initially screened 579 potential publications related to *L. edodes* peptides or polysaccharides. Following rigorous abstract screening and full-text evaluations, combined with relevant information from the HERB database, we ultimately compiled 25 *L. edodes* peptide sequences and 14 *L. edodes* polysaccharides from 66 publications and the HERB database (detailed information shown in Table 1). This provides a reliable data foundation for subsequent network pharmacology analysis and mechanism-of-action research. The number of amino acids in these peptides varied from 2 to 11, and there were 24 peptides having 2 to 7 amino acids, while 1 peptide contained 11 amino acids. The polysaccharide components primarily included D-galactose, D-glucose, D-xylose, D-mannose, L-arabinose, L-rhamnose, and β-glucan.

### 3.2. Target Prediction Analysis of L. edodes Peptides and Polysaccharides

In order to enhance the coverage and reliability of target prediction, a collaborative prediction based on a multi-database strategy was employed to systematically identify potential functional targets for *L. edodes* peptides and polysaccharides (Supplementary Figure S1). Specifically, a target prediction of *L. edodes* peptides using PharmMapper initially resulted in 3080 human protein targets. After elimination of duplicates, 322 targets were retained. Simultaneously, the SwissTargetPrediction database was applied to the same set of peptides, yielding 1870 targets. After removing duplicates, 343 targets remained. Lastly, target prediction of *L. edodes* polysaccharides using the SwissTargetPrediction database initially resulted in 267 targets. After removing duplicates, 47 targets were retained. Integrating and further deduplicating the *L. edodes* peptide and polysaccharide targets from these databases yielded 621 valid, unique human protein targets. They serve as potential targets for *L. edodes* peptides and polysaccharides to exert their anti-T2DM effects, which will be elucidated in subsequent studies.

### 3.3. Systematic Identification of Therapeutic Targets for T2DM

A joint search approach using multiple databases resulted in the procurement of disease targets relevant to T2DM. Searches were conducted using the keywords “T2DM”, “type 2 diabetes mellitus”, and “Diabetes Mellitus, Type 2” in the OMIM, TTD, DrugBank, and GeneCards (relevance score ≥10) databases. Preliminary search results yielded 184, 99, 107, and 8606 targets from OMIM, TTD, DrugBank, and GeneCards, respectively. All initially identified targets were integrated and systematically deduplicated, ultimately yielding 8708 independent T2DM-related targets. This provides the basis for subsequent analysis.

### 3.4. PPI Network Analysis on the Common Targets of L. edodes Peptides and Polysaccharides Against T2DM

**Figure 2:**
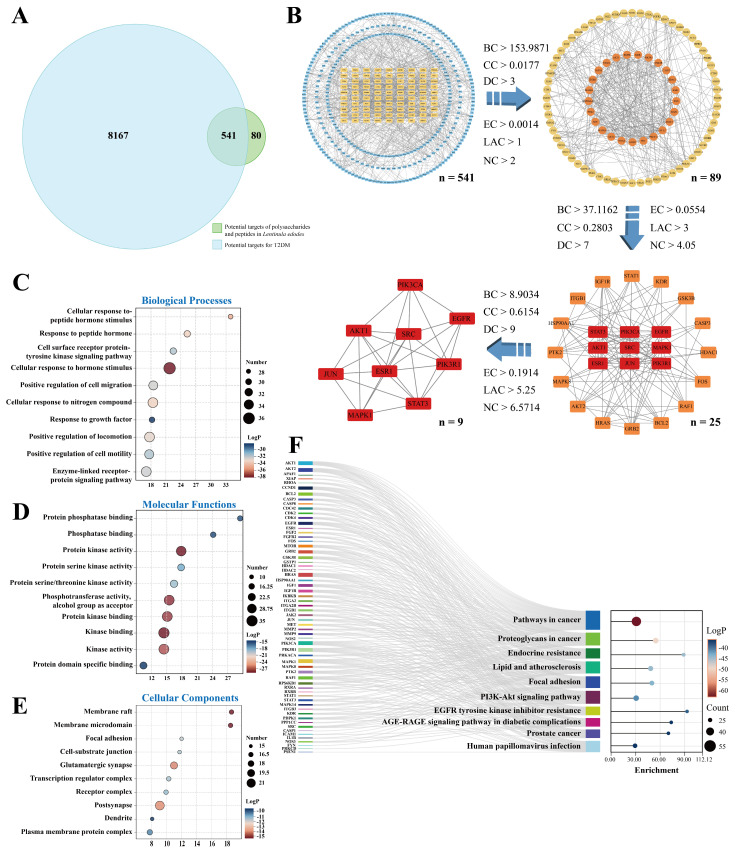
Network pharmacology analysis for the mechanism of *L. edodes* peptides and polysaccharides in treating T2DM. (**A**) Venn diagram showing the intersection of target proteins for *L. edodes* peptides and polysaccharides with T2DM disease targets. Among the 621 *L. edodes* peptide- and polysaccharide-related targets screened from databases and the 8707 T2DM-related targets, 541 common targets were identified. (**B**) Screening process and criteria for identifying core targets in the interaction network between *L. edodes* peptides/polysaccharides and T2DM. (**C**–**E**): Gene Ontology (GO) enrichment analysis of *L. edodes* peptides and polysaccharides for potential targets against T2DM. (**F**): KEGG enrichment analysis of potential anti-T2DM targets for *L. edodes* peptides and polysaccharides.

The 621 targets related to the *L. edodes* peptides and polysaccharides, identified through the abovementioned screening steps, were intersected with the 8708 T2DM-related nodes, obtaining 541 common targets (Figure 2A). The PPI network was created by submitting the intersecting target nodes to the STRING database, with the species restricted to “Homo sapiens” and a minimum required interaction score of 0.900, which is the highest value. Then the result of the STRING analysis was exported in the form of a TSV file and then imported into the Cytoscape 3.10.3 software. Iterative topological analysis was performed using the CytoNCA 2.1.6 plugin. Six topological parameters, namely BC, CC, DC, EC, LAC, and ND, were measured for each target, and key targets with all metrics above the median were picked (Figure 2B). Ultimately, we identified nine core targets—ESR1, MAPK1, AKT1, SRC, EGFR, STAT3, JUN, PIK3CA, and PIK3R1—for subsequent in-depth investigation into their underlying molecular mechanisms.

### 3.5. Functional Enrichment Analysis of Core Targets

**Figure 3:**
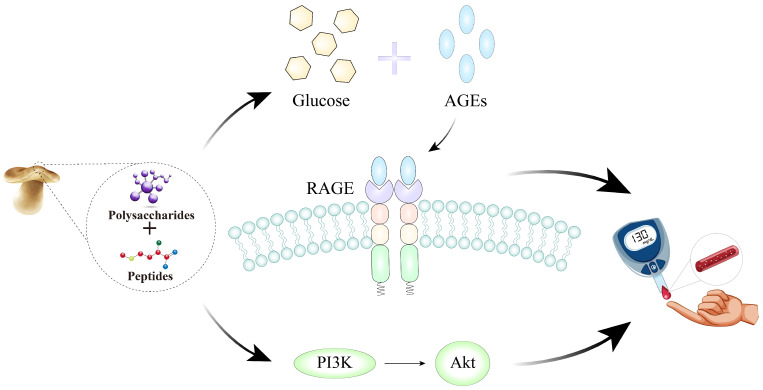
Possible mechanisms of *L. edodes* peptides and polysaccharides in improving T2DM complications via the PI3K-Akt signaling pathway and the AGE-RAGE signaling pathway in diabetic complications.

Based on the 89 targets identified through initial screening, the GO enrichment analysis revealed significant enrichment entries across all three ontology categories: BP, MF, and CC. Within the BP category, targets were predominantly enriched in positive regulation of biological processes, responses to hormone stimulation, and multiple signal transduction pathways (Figure 2C). In the MF category, significantly enriched functions included protein kinase activity, kinase activity, protein serine/threonine kinase activity, and protein phosphatase binding (Figure 2D). In CC analysis (Figure 2E), targets were significantly enriched in structures such as the membrane microdomain, membrane raft, glutamatergic synapse, and postsynapse. Further KEGG pathway analysis (*p* < 0.05, Figure 2F) revealed significant enrichment of these targets in the PI3K-Akt signaling pathway, EGFR tyrosine kinase inhibitor resistance, and the AGE-RAGE signaling pathway in diabetic complications. These pathways are important for glucose and insulin resistance regulation. As shown in Figure 3, the enrichment results suggest that *L. edodes* peptides and polysaccharides may improve T2DM through multiple pathways, primarily by regulating insulin signaling (PI3K-Akt) and inflammatory responses (AGE-RAGE).

### 3.6. Molecular Docking of L. edodes Peptides and Polysaccharides with Target Molecules

**Figure 4:**
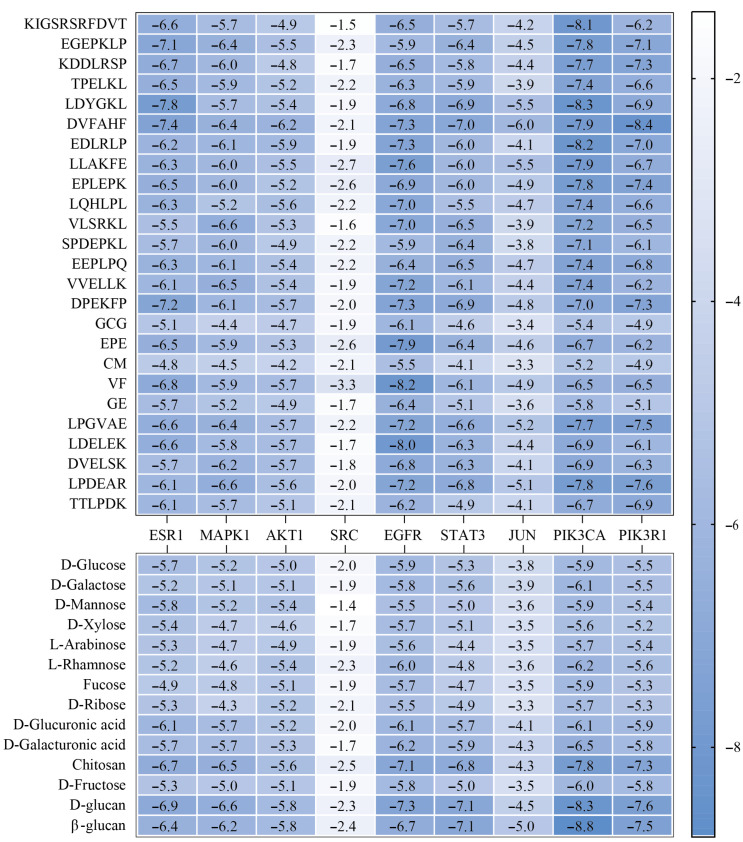
Binding affinity between *L. edodes* peptides and polysaccharides (vertical axis) with core target proteins (horizontal axis). Color intensity corresponds to binding energy scores (kcal/mol), ranging from −1.4 (weak binding, white) to −8.8 (strong binding, blue).

**Figure 5:**
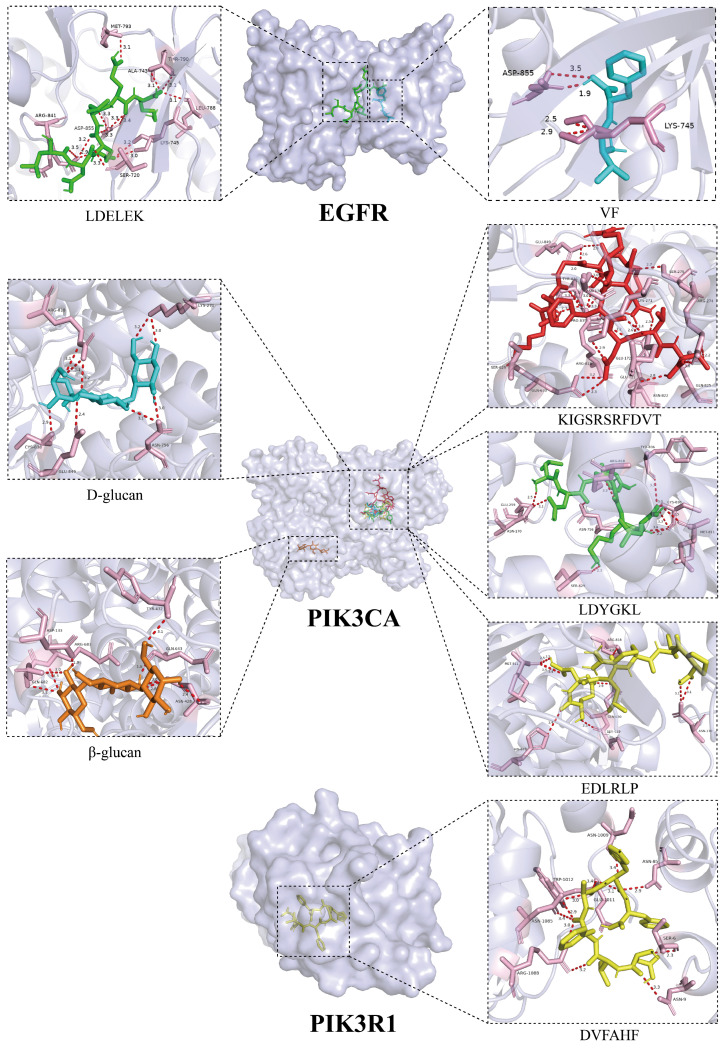
Binding modes of peptides VF and LDELEK with EGFR; binding modes of peptides KIGSRSRFDVT, LDYGKL, and EDLRLP, as well as polysaccharides D-glucan and β-glucan, with PIK3CA; binding mode of peptide DVFAHF with PIK3R1 (The light purple regions indicate target proteins, while the pink regions represent amino acid residues within the target proteins that interact with ligands (*L. edodes* peptides and polysaccharides). Ligands are displayed in multiple colors: green, blue, red, orange, and yellow structures represent different polysaccharides or peptides, respectively. The red dashed lines in the figure indicate hydrogen bond interactions formed between ligands and target residues).

To further investigate the interaction mechanisms between both *L. edodes* peptides and polysaccharides and their core targets, we conducted molecular docking analyses between the nine core targets identified in previous screening and 25 *L. edodes* peptides along with 14 *L. edodes* polysaccharides (Figure 4). The results revealed strong binding affinities between peptides VF and LDELEK and the target EGFR, corresponding to binding energies of −8.2 kcal/mol and −8.0 kcal/mol, respectively. Additionally, peptides KIGSRSRFDVT, LDYGKL, and EDLRLP, along with polysaccharides D-glucan and β-glucan, exhibited significant interactions with the target PIK3CA, with binding energies of −8.1 kcal/mol, −8.3 kcal/mol, −8.2 kcal/mol, −8.3 kcal/mol, and −8.8 kcal/mol, respectively. Simultaneously, the peptide DVFAHF also exhibited significant binding with the target PIK3R1, with a binding energy of −8.4 kcal/mol (Figure 5).

## 4. Discussion

With the continuous rise in global incidence and mortality rates, T2DM has become a major public health challenge [62,63,64,65]. Although first-line clinical drugs like metformin and sulfonylureas are widely used, they still carry significant side effects [66]. Therefore, developing novel therapeutic strategies holds urgent clinical significance. Recently, the application of diet intervention in the prevention and/or management of T2DM has been steadily increasing [67], gradually become an important component of comprehensive T2DM management.

As a kind of large edible and medicinal fungus, *L. edodes* exhibits diverse therapeutic potential [20,49,68,69]. Accumulating evidence shows that both the peptides and polysaccharides of *L. edodes* have potential efficacy for blood glucose regulation and for improving T2DM [26,70,71]. However, the synergistic mechanism between these two classes of bioactive components remains unexplored, necessitating systematic research for in-depth investigation.

In order to investigate the synergistic mechanisms of *L. edodes* peptides and polysaccharides against T2DM, this study first retrieved *L. edodes* peptides and polysaccharides from databases and predicted their potential targets. The analysis revealed 541 common interaction targets with T2DM targets. Among these overlapping targets, the majority are involved in biological processes including insulin signaling pathways and inflammatory responses. Second, we built a protein interaction network for the common targets through the STRING database and performed topological analysis using the Cytoscape 3.10.3 software. This finally identified nine core target genes: ESR1, MAPK1, AKT1, SRC, EGFR, STAT3, JUN, PIK3CA, and PIK3R1. Extensive research shows that these core genes mainly participate in two ways in the pathogenesis of T2DM.

(i) Regulation of insulin signaling: The PI3K/Akt signaling pathway, as a crucial downstream signaling hub of the insulin receptor, has been identified to play significant roles in regulating glucose metabolism and glycogen synthesis [72]. Among these, PIK3CA and PIK3R1, as members of the PI3K family, can activate AKT (e.g., AKT1), which in turn modulates various downstream substrates through serine and/or threonine phosphorylation, thereby influencing cellular metabolic functions [73]. ESR1 (encoding ERα) not only activates insulin signaling independently of IRS1 and IRS2 via the E2-ERα pathway but also upregulates endogenous IRS1 expression in breast cancer cells, thereby modulating glucose homeostasis and insulin sensitivity [17]. SRC, as a non-receptor tyrosine kinase, can directly modulate the function of the PI3K-Akt signaling pathway, contributing to the promotion of glucose metabolism [74,75]. Likewise, EGFR activation enhances the PI3K/Akt signaling pathway, hence stimulating glucose flow and glucose flow-dependent uptake [75]. Experiments further indicate that STAT3 phosphorylation significantly upregulates mRNA levels of cytokine-regulated inhibitor 3 (SOCS3). As a kind of negative regulator, SOCS3 has the ability to efficiently suppress the phosphorylation of the PI3K/Akt signaling pathway, thus causing insulin resistance in the liver by reducing the sensitivity to insulin [76].
(ii) Modulation of inflammatory responses: Many studies have implicated chronic inflammation in the etiology and progression of T2DM [77,78,79]. For instance, the MAPK family includes a critical component, MAPK1, which plays a pivotal role in inflammatory development. Oxidative stress and advanced glycation end products (AGEs) activate MAPK signaling during diabetic states, leading to the secretion of inflammatory mediators and promoting T2DM [80]. In addition, JUN promotes the production of inflammatory factors, reducing hepatic insulin sensitivity and thereby acting to promote insulin resistance in T2DM [77,81].

Then, 89 targets identified from initial screening were subjected to GO and KEGG enrichment analyses. The outcome of the KEGG pathway enrichment analysis indicated that these targets were found to be significantly enriched in endocrine resistance, lipid and atherosclerosis, the PI3K-Akt signaling pathway, EGFR tyrosine kinase inhibitor resistance, and the AGE-RAGE signaling pathway in diabetic complications. The PI3K-Akt signaling pathway is essential for normal metabolism in target tissues, with key functions including regulation of glucose metabolism. Impairment of this pathway results in developing insulin resistance, which subsequently progresses into T2DM [82]. Firstly, this pathway triggers downstream serine/threonine kinases, promoting the translocation of GLUT4 transporters to the cell membrane from the cytoplasm. Thus, it promotes a significant increase in glucose uptake by cells, thereby lowering blood glucose concentration [72]. Second, it also alleviates T2DM by modulating the activity of GSK-3β to improve glycogen accumulation [83]. Third, the PI3K-Akt signaling pathway upregulates the expression of PFKFB3 to promote glycolysis [84,85], playing a positive role in T2DM [75]. Additionally, elevated circulating glucose levels associated with insulin resistance tend to bind with proteins and lipids to form AGEs. These substances have also been cited to be among the major factors responsible for the evolution and continuous development of diabetic pathologies [70]. By binding to RAGE (receptor for AGEs), they can further modulate the NF-κB and PI3K/Akt signaling pathways, influencing T2DM progression through both inflammatory and glucose metabolic pathways [86,87].

Next, the molecular docking method was applied to systematically analyze the binding characteristics of 25 peptides and 14 polysaccharides with nine core targets to illustrate the interaction mechanisms of *L. edodes* peptides and polysaccharides with the core targets. The results indicated that there were strong binding affinities between the peptides VF (−8.2 kcal/mol) and LDELEK (−8.0 kcal/mol) with the EGFR target; peptides KIGSRSRFDVT (−8.1 kcal/mol), LDYGKL (−8.3 kcal/mol), and EDLRLP (−8.2 kcal/mol), along with polysaccharides D-glucan (−8.3 kcal/mol) and β-glucan (−8.8 kcal/mol), showed significant interaction with the PIK3CA target. The peptide DVFAHF also exhibited high binding affinity with the PIK3R1 target, with a binding energy of −8.4 kcal/mol.

Therefore, based on the results of integrating molecular docking and KEGG enrichment analysis, this study suggests that *L. edodes* peptides and polysaccharides may improve T2DM by acting on multiple targets and pathways. Specifically, the strong binding of peptides KIGSRSRFDVT, LDYGKL, and EDLRLP, along with polysaccharides D-glucan and β-glucan, to PIK3CA and the high binding affinity of peptide DVFAHF to target PIK3R1 suggest that these components may exert anti-T2DM effects by regulating the PI3K-Akt signaling pathway. Meanwhile, the AGE-RAGE pathway can modulate the PI3K/Akt signaling pathway and the NF-κB pathway, thus jointly affecting T2DM through glucose metabolism and inflammation.

Several limitations exist for the current study. Firstly, many *L. edodes* peptide and polysaccharide components may remain inadequately explored and identified at present, preventing this research from covering these potentially relevant constituents. Secondly, although multiple databases were integrated for target prediction to increase the coverage and reliability of the results, incomplete information on *L. edodes* peptides, polysaccharides, and T2DM-related targets in existing databases may have resulted in the exclusion of certain key targets or pathways from analysis [29,88]. Finally, the potential targets and pathways derived from network pharmacology analysis remains to be verified by subsequent experiments [28,89].

## 5. Conclusions

This study comprehensively leveraged network pharmacology and molecular docking methods to elaborate the potential mechanisms of *L. edodes* peptides and polysaccharides in alleviating T2DM through multi-target and multi-pathway synergistic effects. Results indicate that *L. edodes* peptides and polysaccharides exhibit strong binding affinities with the core targets, including EGFR, PIK3CA, and PIK3R1. This suggests that these peptides and polysaccharides may exert a synergistic effect to enhance insulin sensitivity and mitigate glucose metabolic disorders by regulating key pathways like PI3K-Akt and inflammation mediated by AGE-RAGE. This study provides theoretical support for the use of *L. edodes* peptides and polysaccharides as a potential intervention strategy against T2DM while also guiding future *L. edodes*-based drug and functional food development research.

## Figures and Tables

**Table 1:** The detailed information of *L. edodes* peptides and polysaccharides.

Peptides/Polysaccharides	Number	Sequence/Classification	References
Peptides	1	KIGSRSRFDVT	[31]
	2	EGEPKLP	[26]
	3	KDDLRSP	
	4	TPELKL	
	5	LDYGKL	
	6	DVFAHF	
	7	EDLRLP	
	8	LLAKFE	
	9	EPLEPK	
	10	LQHLPL	
	11	VLSRKL	
	12	SPDEPKL	
	13	EEPLPQ	
	14	VVELLK	
	15	DPEKFP	
	16	GCG	[32]
	17	EPE	
	18	CM	
	19	VF	
	20	GE	
	21	LPGVAE	[33]
	22	LDELEK	
	23	DVELSK	
	24	LPDEAR	
	25	TTLPDK	
Polysaccharides	1	D-glucose	[34,35,36,37,38,39,40,41,42,43,44]
	2	D-galactose	[35,36,37,38,39,40,41,43]
	3	D-mannose	[35,36,38,39,40,41,43,45]
	4	D-xylose	[35,36,39,42,43,46]
	5	L-arabinose	[36,37,38,41,43,47]
	6	L-rhamnose	[36,38,41,42,43,47]
	7	Fucose	[41,43,48]
	8	D-ribose	[49]
	9	D-glucuronic acid	[42,50]
	10	D-galacturonic acid	[42]
	11	Chitosan	[51]
	12	D-fructose	[50]
	13	D-glucan	[52]
	14	β-glucan	[27,53,54,55,56,57,58,59,60,61]

## Data Availability

The original contributions presented in the study are included in the article/Supplementary Materials. Further inquiries can be directed to the corresponding author.

## Supplementary Materials

The following supporting information can be downloaded at https://www.mdpi.com/article/10.3390/foods15030453/s1, Table S1: The specific parameters for molecular docking; Figure S1: Summary of target sites for *L. edodes* peptides and polysaccharides.
