# Supplementary material for: Synergistic Mechanistic Insights into Anti-T2DM Benefits of Lentinula edodes: A Peptide- and Polysaccharide-Based Network Pharmacology and Molecular Docking Study
Source: Foods. 2026 Jan 27;15(3):453. doi: 10.3390/foods15030453 (PMC12896406; doi:10.3390/foods15030453)

Supplementary Table S1: The specific parameters for molecular docking.

**Receptor**	**Ligand**	**Center**	**Size**
ESR1 (4XI3)	Peptides	Center_X = -1.3<br>Center_Y = -23.831<br>Center_Z = 0.341	Size_X = 96.95<br>Size_Y = 96.95<br>Size_Z = 96.95
	Polysaccharides		
MAPK1 (7NR9)	Peptides		Size_X = 70.35<br>Size_Y = 70.35<br>Size_Z = 70.35
	Polysaccharides		
AKT1 (1H10)	Peptides	Center_X = 21.707<br>Center_Y = 14.463<br>Center_Z = 9.91	Size_X = 40.5<br>Size_Y = 38.25<br>Size_Z = 47.25
	Polysaccharides		
SRC (4M4Z)	Peptides		Size_X = 47.25<br>Size_Y = 32.25<br>Size_Z = 47.25
	Polysaccharides		
EGFR (8A27)	Peptides (from KIGSRSRFDVT to EDLRLP)		Size_X = 64.75<br>Size_Y = 55.5<br>Size_Z = 64.75
	Peptides (from LLAKFE to TTLPDK) + Polysaccharides	Center_X = 10.044<br>Center_Y = -6.509<br>Center_Z = -16.145	
STAT3 (6NJS)	Peptides	Center_X = -2.257<br>Center_Y = 19.47<br>Center_Z = 24.491	Size_X = 110.95<br>Size_Y = 109.19<br>Size_Z = 110.95
	Polysaccharides		
JUN (1JNM)	Peptides		Size_X = 89.95<br>Size_Y = 89.95<br>Size_Z = 89.95
	Polysaccharides		
PIK3CA (8EXL)	Peptides		
	Polysaccharides		
PIK3R1 (2IUI)	Peptides	Center_X = 23.293	Size_X = 59.85

	Polysaccharides	Center_Y = 0.53<br>Center_Z = 14.864	Size_Y = 59.85<br>Size_Z = 59.85

Figure S1. Summary of target sites for *L. edodes* peptides and polysaccharides.

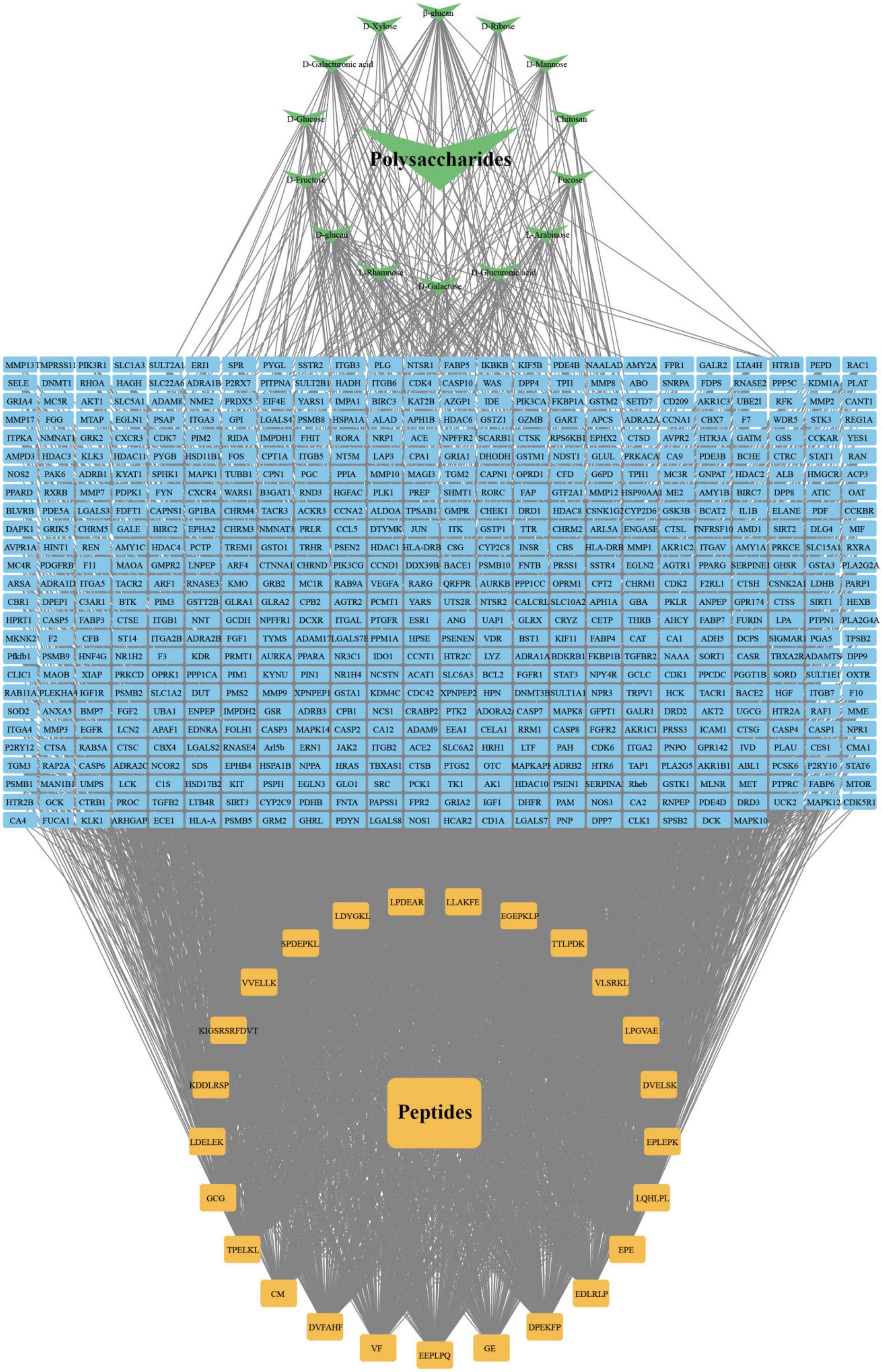

Supplement: Supplementary file 1 [file foods-15-00453-s001.zip › foods-4077167-supplementary.pdf]
